# How Schroth Therapists Vary the Implementation of Schroth Worldwide for Adolescents with Idiopathic Scoliosis: A Mixed Methods Study

**DOI:** 10.3390/jcm12186063

**Published:** 2023-09-19

**Authors:** Rosemary Marchese, Emre Ilhan, Verity Pacey

**Affiliations:** 1 ScoliCare, Sydney 2217, Australia; rosemary.marchese@scolicare.com; 2 Department of Health Sciences, Macquarie University, Sydney 2109, Australia; emre.ilhan@mq.edu.au

**Keywords:** scoliosis, Schroth, idiopathic scoliosis, adolescent, exercises

## Abstract

(1) Background: Schroth is a type of physiotherapeutic scoliosis specific exercise (PSSE) prescribed to adolescents with idiopathic scoliosis (AIS). Studies have investigated the effectiveness of Schroth but are yet to elucidate how Schroth is applied clinically and the factors that influence their prescription. (2) Methods: A mixed methods design was used comprising an anonymous survey and semi-structured interviews of Schroth therapists who treated AIS and who were publicly listed on the Barcelona Scoliosis Physical Therapy School or the International Schroth 3-dimensional Scoliosis Therapy School websites. The survey included 64 questions covering demographics, session and treatment characteristics, and whether therapists included other treatment modalities in their clinical practice. A convenience sample of survey participants were invited to participate in a semi-structured interview to further explore the factors that influenced their prescription of Schroth for AIS. Results from the survey were analyzed descriptively (n, %), whereas inductive thematic analysis was used for the interviews. (3) Results: of the 173 survey respondents (18% response rate), most were from Europe and North America (64.0%), female (78.6%), physiotherapists (96.0%), and worked in private settings (72.3%). Fifty-two per cent of participants used other types of PSSE as an adjunct to Schroth, the Scientific Exercise Approach to Scoliosis (SEAS) being the most frequently used (37.9%). Non-PSSE methods were used ‘at some point’ as an adjunct by 98.8% of participants, including massage and other soft tissue techniques (80.9%), Pilates (46.6%), and Yoga (31.5%). The Schroth techniques used by all survey respondents included breathing and pelvic corrections. Seven participants were interviewed, but data saturation was achieved after only four interviews. Thematic analysis revealed four, inter-related broad themes describing the factors that influenced Schroth prescription for AIS: (1) the adolescent as a whole, including physical, emotional and mental characteristics, and patient goals, (2) family, including parent relationship with the adolescent and the motivation of parents in regard to Schroth, (3) the systems within which the treatment was being offered, such as vicinity to the clinic and the presence of financial insurance support, and (4) therapist characteristics, such as their training and experience. (4) Conclusions: Schroth therapists worldwide use a variety of adjunctive methods to treat AIS. Therapists prescribing Schroth exercises to AIS consider the complex interplay of intra-, inter- and extra-personal factors in clinical practice. These considerations move beyond the three components of evidence-based practice of research, patient preferences, and clinical expertise, towards a systems-based reflection on exercise prescription.

## 1. Introduction

Idiopathic scoliosis is diagnosed when all other causes of scoliosis have been excluded [1]. Adolescent idiopathic Scoliosis (AIS) affects 2% to 3% of the population, of which only 0.3% to 0.5% of affected patients will have a curvature of >20 degrees, the curve magnitude at which treatment is generally recommended [1,2]. AIS is diagnosed from 10 to 17 years of age [2] and progression risk is most heightened at the beginning of puberty [2,3,4]. Years ago, research confirmed that vertebral growth is modulated by loading, as described by the Heuter–Volkmann principle [5].The need for more research was suggested to quantify this relationship to permit a better design of conservative treatment of spinal deformity during the adolescent growth spurt [5]. Conservative treatment for AIS may include regular clinical evaluation, physiotherapeutic scoliosis specific exercises (PSSE) and bracing, before surgery is recommended for larger curves [2]. The term PSSE was introduced and defined by the International Society of Scoliosis and Orthopedic and Rehabilitation Treatment (SOSORT) to include the following: (1) autocorrection in three dimensions, (2) stabilization of the corrected posture, (3) integration into activities of daily living, and (4) patient education [2]. Multiple PSSE training schools claim to alter the asymmetric loading [5,6,7] that occurs with AIS. The aim is to promote spinal and trunk alignment using specific exercises to reduce or stop the asymmetric loading and potentially stopping scoliosis progression [7].

PSSE is taught to therapists around the world using methods from various teaching schools [7]. The specific characteristics of each PSSE program and the factors influencing programming decisions taught by varying schools are not always explicit in the literature [8], which may create challenges for the application of the methods in clinical practice and research studies [9]. When details are provided within the literature, specifics about exercise dosage, such as frequency and duration, differ considerably between studies [10]. No study has described how therapists trained in PSSE worldwide are applying therapy to patients with AIS. Furthermore, no studies that investigate the benefit of PSSE for AIS have justified the PSSE prescription in relation to the extent of the present spinal and postural deformity in the AIS treatment groups. This may go some way to explaining why only 21% of respondents to a survey of Scoliosis Research Society members prescribe or refer for PSSE for AIS [11].

Schroth, a type of PSSE, is a complex exercise treatment, taught by multiple therapists across multiple continents. Understanding how Schroth is being applied to AIS worldwide once therapists have completed training and the key factors that influence the success of exercise treatment for AIS, has not been elucidated to date [8]. The Schroth method is one of the most frequently studied and used PSSE methods to date [12]. However, studies have generally focused on comparisons of the treatment against other conservative treatments rather than exploring the impact that a variation in Schroth prescription (e.g., exercise, intensity, repetitions) has for various amounts of curve deformity. Therefore, to increase the understanding of how Schroth is applied into current clinical practice, this study aimed to explore how therapists are applying the Schroth method for AIS, and the factors that influence their prescription in clinical practice worldwide. To do this, the survey aimed to explore the following research questions: (1) Is the application of Schroth consistent across trained therapists treating AIS? and (2) Do therapists combine Schroth exercises with other treatment methods to treat AIS? The results of the survey were then used by the researchers to help formulate open-ended interview questions to explore the following: (1) What are the factors that influence prescription of Schroth for AIS worldwide? and (2) What is the rationale for the treatment programming and application? Together, the results of these two parts of the study were used to answer the question ‘How Schroth therapists vary the implementation of Schroth worldwide for adolescents with idiopathic scoliosis’ in the form of a mixed methods study.

## 2. Materials and Methods

### 2.1. Study Design

This study used a mixed methods design comprising a survey and semi-structured interviews. The study is reported here according to A Consensus Based Checklist for Reporting of Survey Studies (CROSS) (Appendix A) [13] and the Consolidated Criteria for Reporting Qualitative Research Checklist (COREQ) (Appendix B) [14].

### 2.2. Participants

**Figure 1:**
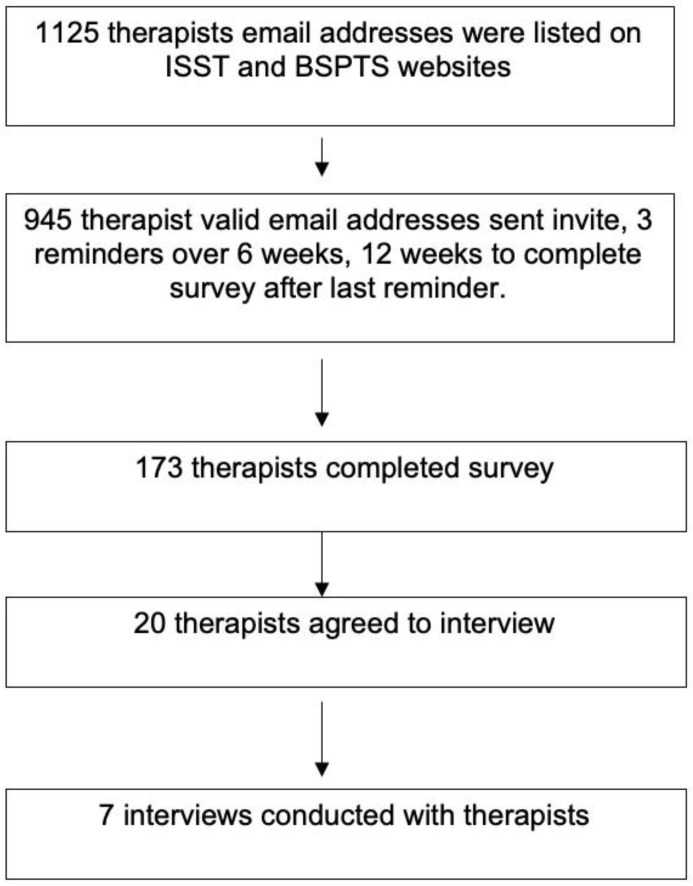
Flowchart of participant inclusion through the study: Part 1, a survey of Schroth therapists’ application of Schroth for adolescents with idiopathic scoliosis (AIS), Part 2, an interview of Schroth therapists to explore the factors that influence their application of Schroth worldwide.

Schroth therapists were eligible for the survey and semi-structured interviews if they were (1) proficient in English, (2) currently treating AIS using Schroth, (3) listed on either or both of the website directories for Barcelona Scoliosis Physical Therapy School [15] or International Schroth 3-dimensional Scoliosis Therapy (ISST) [16]. A search of the therapist directories yielded 945 valid emails to which invitations to participate were sent (Figure 1).

### 2.3. Data Collection

#### Survey

Schroth therapists were invited via email to participate in an anonymous online survey (Appendix C) via REDCap (over twelve weeks). Survey questions were developed using a comprehensive list of Schroth treatment techniques and exercise options listed in course content [17] and a published protocol [18]. The survey included 64 questions covering demographics, session characteristics such as duration, treatment characteristics such as session duration, and exercises used in Schroth, along with the inclusion of other PSSE, non-PSSE, and any other exercises that they used within clinical practice.

### 2.4. Interviews

All survey participants were invited to provide their details in an unlinked form via REDCap to receive an invitation to participate in a semi-structured interview. Open-ended questions (Appendix D) were developed following analysis of survey responses and were based around a basic hypothetical scenario of an adolescent with idiopathic scoliosis, which aimed to elicit the following information: (1) factors that influenced the prescription of Schroth therapy in AIS, and (2) rationale for treatment programming and application. The interviews were conducted, recorded, and transcribed by RM via Zoom. RM is an Australian female physiotherapist with 22 years of experience, who trained and practiced in the BSPTS method for 6 years and is completing a Master of Research in Australia. The transcription was checked by RM and EI and then emailed for respondent validation. EI is a physiotherapist with 6 years of experience and qualitative research experience.

### 2.5. Data Analysis

#### Survey

The survey was analyzed using descriptive statistics in Microsoft Excel for Mac V16.58. The data were grouped into (1) demographics, (2) general characteristics of the program, (3) frequency and duration of treatment, (4) SOSORT, PSSE, and non-PSSE features, (5) cues, corrections, and techniques, (6) strategies to adjust exercise difficulty, (7) support material, (8) factors affecting prescription, and (9) compliance monitoring. Response frequencies were calculated from valid responses with missing responses stated as a percentage of total participants.

### 2.6. Interview

To minimize the risk of bias given that RM is trained and certified in BSPTS, EI, who does not have specific scoliosis or Schroth experience or training, was also involved in the inductive thematic analysis of the interview data. The analysis took an iterative approach, as described by Braun and Clarke (2006), where new codes were independently developed, cross-checked by two members (RM and EI), and once agreement was reached, the codes were grouped into themes to ensure authenticity with the experience of the participants [19]. Any disagreements were resolved through discussion with a third investigator (VP). The point of data saturation was monitored during recruitment. Saturation was reached when no new themes emerged from interviews. Interviews to explore the factors that influenced Schroth prescription were conducted between August and October 2021. The social ecological model of health was used as the lens to interpret and triangulate the findings [20].

## 3. Results

### 3.1. Survey

#### 3.1.1. Survey Participant Demographics

One hundred and seventy-three (18% response rate) surveys were completed (Figure 1). Most participants were from Europe and North America (64.0%), female (78.6%), physiotherapists (96.0%), and worked in private settings (72.3%) (Table 1). Of the 28 languages identified, English was most commonly used in clinical practice (44.0%).

#### 3.1.2. Characteristics of Schroth Program

A majority (87.3%) of participants reported being ‘very’ or ‘somewhat’ familiar with these SOSORT guidelines (Table 2). Most participants reported using the recommended components of PSSE according to SOSORT guidelines [2], with 5% ‘rarely’ or ‘never’ following these recommendations (Table 3).

**Figure 2:**
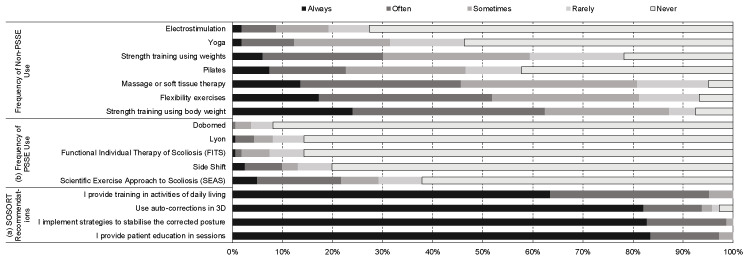
The characteristics and training background of Schroth therapists and their application of Schroth for adolescents with idiopathic scoliosis including. This figure shows the proportion of Schroth therapists that implement the recommendations for physiotherapeutic scoliosis specific exercises (PSSE) as per ^a^ SOSORT guidelines. ^a^ SOSORT is the International Society of Scoliosis Orthopedic and Rehabilitation Treatment. ^b^ PSSE is physiotherapeutic scoliosis specific exercises.

Other types of PSSE as an adjunct to Schroth were used by 52.5% of participants, SEAS being the most popular (Figure 2). Non-PSSE methods were used ‘at some point’ as an adjunct by 98.8% of participants, where massage and other soft tissue techniques were the most popular (80.9%) followed by Pilates (46.6%) and Yoga (31.5%) (Figure 2).

**Figure 3:**
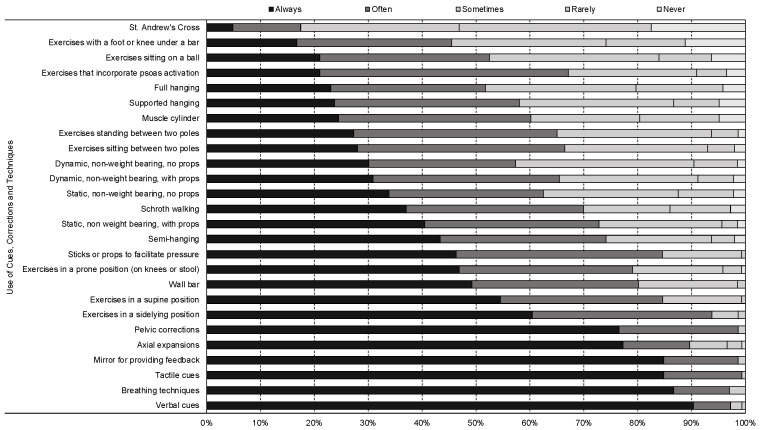
Cues, corrections, and techniques that are implemented by Schroth therapists worldwide when delivering Schroth exercise prescription to adolescents with idiopathic scoliosis.

**Figure 4:**
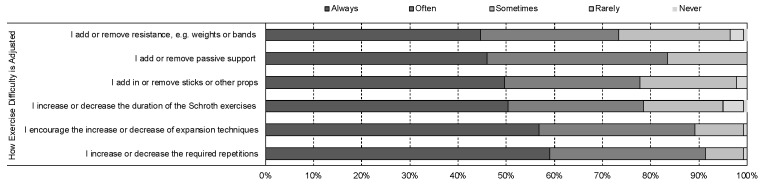
Strategies to adjust exercise difficulty of the exercise program that are implemented by Schroth therapists worldwide when delivering Schroth exercise prescription to adolescents with idiopathic scoliosis.

There was consistent use of Schroth techniques, with low rates of ‘rarely’ or ‘never’ using breathing techniques (0.0%), pelvic corrections (0.0%) or axial expansions (3%) (Figure 3). They also consistently (‘always’ or ‘often’) used verbal (97.2%) and tactile cues (99.3%) when teaching Schroth exercises (Figure 3). The most popular (‘always’ or ‘often’) Schroth exercises were side lying (93.8%) and supine (84.6%) (Figure 3). Exercise difficulty was ‘always’ or ‘often’ adjusted using an increase or decrease in repetitions (91.4%) and/or forces such as those used in expansion techniques (89.2%) (Figure 4).

Most participants conducted more than five sessions per week (74.5%) with a majority (77.8%) using Schroth for AIS for five or more hours per week (Table 3). The majority of sessions lasted 60 min per session (60.8%) (Table 3). Most (94.2%) recommended sport participation, and all (100.0%) provided a home exercise program, although the frequency of recommendation did vary (Table 2).

#### 3.1.3. Factors Affecting Exercise Prescription

**Figure 5:**
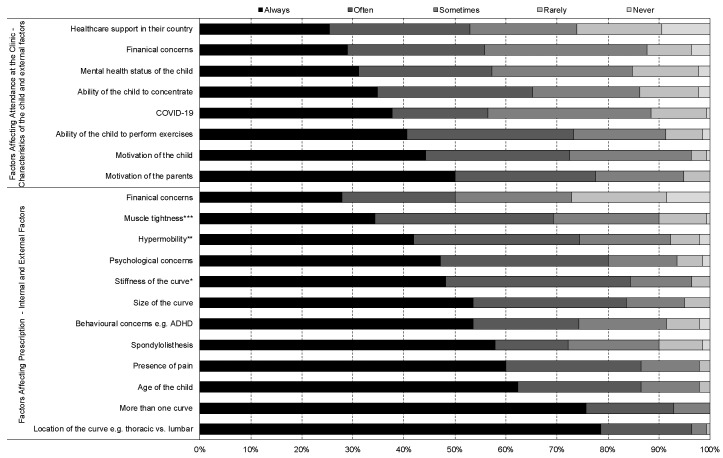
Factors affecting prescription and attendance at the clinic when Schroth therapists are delivering Schroth exercise prescription to adolescents with idiopathic scoliosis. * Stiffness of the curve, as defined by ability of the hump to reduce in size with particular movements. ** Hypermobility was defined by Beighton’s score 6 or more out of 9. *** Muscle tightness was described as a positive Thomas test or straight leg raise <90 degrees. ADHD is attention deficit hyperactivity disorder.

Participants reported that exercise prescription was ‘always’ or ‘often’ or ‘sometimes’ (97.1%) determined by the location of the curve (Figure 5) and the presence of more than one curve (100.0%) (Figure 5). Attendance at the clinic was also perceived by participants to be affected by the motivation of the parents (94.9%) and the adolescent (96.4%) (Figure 5).

**Figure 6:**
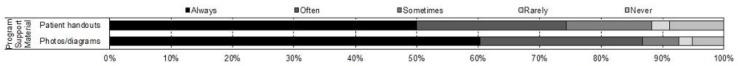
Support material provided to adolescents with idiopathic scoliosis by Schroth therapists worldwide, implementing Schroth to these patients.

**Figure 7:**
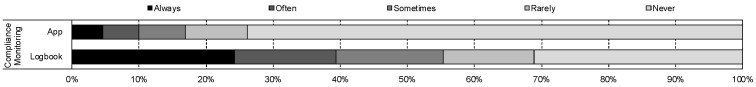
Compliance monitoring techniques used by Schroth therapists worldwide when delivering Schroth to adolescents with idiopathic scoliosis.

Participants ‘always’ or ‘often’ provided education and support to AIS using photos/diagrams (86.8%) (Figure 6) and monitored compliance using a logbook (39.4%) (Figure 7). Performance checklists were only used by a minority (34.6%) of participants and most (76.1%) had designed the checklist themselves (Table 3).

### 3.2. Interview

#### 3.2.1. Interview Participant Demographics

The interview participants were from Europe (n = 3), North America (n = 2), and South America (n = 2). Two had BSPTS-only training, one had ISST-only training, and four had both. Two were previous colleagues of RM but had no prior involvement in the project. EI did not know any participants. All participants had between two and nine years of Schroth experience treating AIS.

#### 3.2.2. Thematic Analysis

Data saturation occurred by the fourth interview; however, three more interviews were conducted to confirm saturation and ensure we achieved representativeness of geographical spread and clinical experience.

Throughout coding, any distinct differences between participants from different regions were considered; however, no specific geographic influences beyond the major underlying themes were noted. Consequently, we decided not to group participants according to location during thematic analysis. Four super-ordinate themes emerged through thematic analysis: (1) adolescent as a whole; (2) family; (3) systems, and (4) therapist (Table 4).

**Figure 8:**
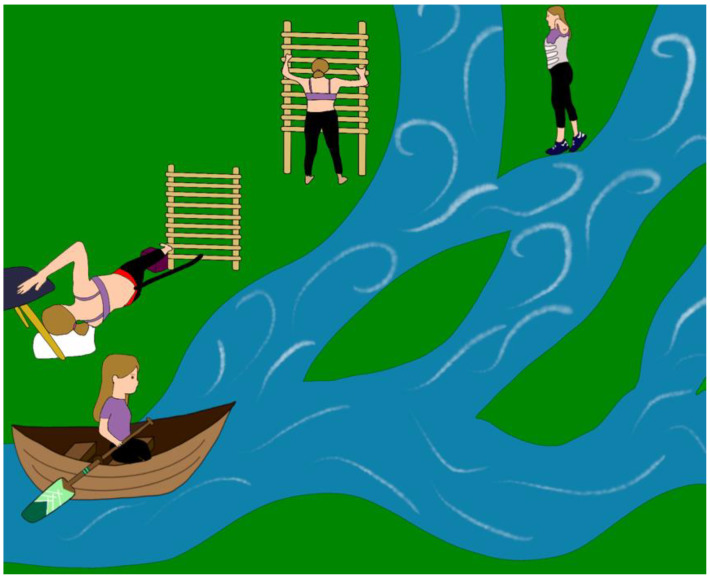
The Schroth journey for the adolescent with idiopathic scoliosis (AIS) can be convoluted and not straightforward. The journey can often change. This figure demonstrates how the adolescent may start off their Schroth exercise prescription along one path, for example with exercises and no bracing, and then that may change where bracing is implemented. Child: the adolescent with idiopathic scoliosis. Boat: the parents/guardians. Paddle: the therapist directing the journey. Water system: the system that needs to be navigated along the journey, which can change (the Schroth journey can be challenging to navigate, and treatment can change with time).

Each theme interacted dynamically with each other, as depicted diagrammatically in Figure 8. Namely, factors that influence Schroth prescription are shaped by parents (the boat) and the adolescent (the child in the boat), who is empowered by the therapist (paddle) to achieve their goals within the specific health systems and contexts (waterway) of the therapy.

## 4. Discussion

Our study found that the application of Schroth in AIS in the clinic is highly variable. The journey may often be arduous which means that the factors affecting Schroth prescription can change over time. The lack of clarity of how Schroth therapy is applied for AIS may be a possible reason for the lack of confidence of Scoliosis Research Society (SRS) members in referring for PSSE for AIS [11]. Furthermore, Schroth therapists use multiple treatment methods beyond BSPTS and ISST methods to treat AIS [17,18]. Treatments applied by Schroth therapists included core components of Schroth, including a variety of cues (e.g., tactile, verbal), corrections (e.g., pelvic corrections, axial expansions), and tools (e.g., various props, mirrors) to deliver and adjust the program [17]. The use of these core components was consistent. However, adaptations were made to suit the patients and context [17,18] and included the use of more peripheral aspects of the treatment, and less often, other PSSE, such as SEAS, and non-PSSE, such as strength training, flexibility, and massage. These reports of large variations in approach by the Schroth therapists in this study are not replicated in the current research studies, with multiple studies focusing simply on exploring the benefits of one approach using Schroth compared with other treatments or no treatment [12,18,21,22]. There is also a lack of description in these studies regarding how the Schroth therapy prescription may vary according to the level of structural deformity in AIS.

In our study, Schroth therapists indicated that intensity is adjusted in multiple ways including a change in repetitions, duration, and adding or removing of props. This variation in the application of Schroth is not well explained in published randomized controlled trials, which can make application into clinical practice difficult [9]. Other factors that add to the complexity of the treatments being applied were the recommendations to participate in sport and integrating treatment into activities of daily living (Figure 2). Both these recommendations are in accordance with the current SOSORT guidelines [2], but how these recommendations are applied in randomized controlled trials has not always been made clear by researchers [9].

The interviews provided an opportunity to explore the factors influencing Schroth prescription in AIS, with four themes identified that interact and influence prescription: (1) the adolescent, (2) the adolescent–parent relationship, (3) the systems, and (4) the therapist, in which Schroth therapy takes place. These themes appear to fit well into the social ecological model of health, with consideration of treatment influencing factors related to the patient and the systems and people (parents and therapist) in their treatment network [20].

The participants described how the experience of the therapist, combined with the characteristics of the child and support from the parents were major influences in the Schroth program. One of the factors influencing attendance at the clinic was affordability and ease of access. Without a good support system from doctors, financial support, and easy access to a Schroth clinic, the frequency of treatment reduced, or telehealth became more frequent. That is, therapists must adapt their programs to suit the state of the systems around them, raising issues of equity and fairness. It is possible that if the adolescent exists within a complex medical system that is not supportive of Schroth, then this creates a variation in accessibility to Schroth treatment and the frequency of supervised sessions reduces. The supervised sessions appear to be important because the completion of a formal Schroth training program with a Schroth instructor has been shown to correlate with an increase in compliance in a home exercise program [23]. It would be useful to determine the level of supervision, system support and accessibility, and motivation strategies required to achieve positive outcomes considering the supervision of Schroth exercises has been noted to be more beneficial than unsupervised Schroth [21]. Overall, the Schroth therapists are having to adapt their programs to suit the systems and therefore no one program of treatment is likely to be the same as another given the varying health systems across the world. It would therefore be useful for medical doctors considering referring AIS to Schroth therapists to know more about how the prescritpion of Schroth is being adapted to suit the curve type and size. For the 78% of surgeons from the SRS surveyed who do not refer AIS for PSSE, the main reasons cited were a lack of supporting research, that PSSE offers no value, and the lack of access to a facility providing PSSE [11].

Despite the fact Schroth therapists in our study commonly incorporated other PSSE and non-PSSE into their treatment plans for AIS, the current research on Schroth is not representative of a mixed approach utilized by more than half of therapists in this study. Even a recent systematic review investigating the effects of the Schroth method compared with core stabilization exercises, and another comparing Schroth and SEAS, included studies compared interventions but were not looking for the effects of combining methods [24,25]. Research has not confirmed the best types of PSSE or PSSE combinations with and without any non-PSSE adjuncts [8].

Future studies should clarify the effect of combining different corrective exercise modalities as well as the most appropriate dose in terms of number and duration of therapeutic sessions [8]. Indeed, future research should explore how combinations of PSSE with and without non-PSSE, as well as other characteristics of the program and level of supervision and compliance, may impact outcomes.

Consideration of the findings will enable future trials to evaluate interventions that are generalizable to the real-world application of Schroth therapy for AIS. For example, the clinical feasibility of interventions worldwide should account for systemic constraints to optimal exercise prescription, including financial barriers and vicinity to a clinic. Future studies could explore the effects of combinations of Schroth with other PSSE and non-PSSE methods for AIS with consideration of the factors influencing prescription described by participants in our study to optimize outcomes for AIS.

### Limitations

This study was strengthened by its mixed methods design, which enabled us to further explore the factors influencing exercise prescription in AIS by Schroth therapists [26]. Although participants in both stages of this study were generally representative of Schroth therapists worldwide, a limitation was that the study was conducted in English, thereby limiting the participation of individuals who could not communicate in English. This may have contributed to the low response rate (18%) for the survey. The study responses were also limited mostly to women. Finally, while we aimed to achieve purposeful sampling for increased representativeness of interviewees, only a small number of participants provided their contact details to be interviewed. This meant we could only achieve a convenience sample. Nevertheless, we were able to reach data saturation during thematic analysis of the interviews.

## 5. Conclusions

This study provides an insight into how Schroth therapists vary the implementation of Schroth worldwide for adolescents with idiopathic scoliosis. Schroth therapists worldwide use a variety of adjunctive methods to treat AIS, including PSSE and non-PSSE methods. The prescription of Schroth is influenced by the adolescent, their parent/s, the therapist, and the systems within which treatment is applied, aligning with a person- and family-centred approach to care. Therapists are more likely to be able to provide more frequent supervised sessions in a supportive health care system, particularly when there is a positive relationship between adolescent and parents. The overall dose and types of exercises prescribed are influenced by the characteristics and motivation of the adolescent and the experience and training of the therapist.

## Figures and Tables

**Table 1:** Demographic characteristics of survey and interview participants.

	Survey n = 173 (% n of Each Category)	Interview n = 7 (% n of Each Category)
Gender		
Female	136 (78.6)	6 (85.7)
Male	37 (21.4)	1 (14.3)
Age		
21–30 years	42 (24.3)	0 (0.0)
31–40 years	64 (37)	2 (28.6)
41–50 years	39 (22.5)	1 (14.3)
51–60 years	24 (13.9)	4 (57.2)
61+ years	4 (2.3)	0 (0.0)
Profession		
Physiotherapist	166 (96)	7 (100.0)
Osteopath	2 (1.2)	0 (0.0)
Chiropractor	1 (0.6)	0 (0.0)
Other	4 (2.4)	0 (0.0)
Location		
North America	55 (32)	2 (28.6)
Europe	55 (32)	3 (42.9)
Asia	43 (24.9)	0 (0.0)
South America	8 (4.6)	2 (28.6)
Australian/New Zealand	6 (3.5)	0 (0.0)
Africa	3 (1.7)	0 (0.0)
United Kingdom	3 (1.7)	0 (0.0)
Type of clinic		
Private	125 (72.3)	6 (85.7)
Hospital	35 (20.2)	1 (14.3)
Other	13 (7.5)	0 (0.0)
Multidisciplinary team ^a^		
Yes	91 (59.5)	6 (85.7)
No	62 (40.5)	1 (14.3)
Language spoken to patients		
English	76 (43.9)	2 (28.6)
Turkish	18 (10.4)	0 (0.0)
Croatian	9 (5.2)	2 (28.6)
Spanish, including Basque and Catalan	9 (5.2)	0 (0.0)
Portuguese	6 (3.5)	2 (28.6)
Other	55 (31.8)	1 (14.3)
Schroth training		
BSPTS ^b^ only	38 (22)	2 (28.6)
ISST ^c^ only	10 (6)	1 (14.3)
Both BSPTS and ISST	125 (72)	4 (57.1)
Level of Training		
Advanced (beyond Level 1 in ISST, BSPTS or both)	127 (76.5)	6 (85.7)
Beginner (Level 1 only in ISST, BSPTS or both)	39 (23.5)	1 (14.3)
Training in other types of PSSE ^d,e^		
Yes	65 (39.2)	4 (57.1)
No	101 (60.8)	3 (42.9)
Schroth Experience ^f^		
<1 year	8 (4.9)	0 (0.0)
2 to <3 years	62 (37.8)	1 (14.3)
3 to <5 years	43 (26.2)	3 (42.9)
5 to <10 years	32 (19.5)	3 (42.9)
10 to 20 years	19 (11.6)	0 (0.0)

^a^ n = 153 due to 20 missing responses. ^b^ BSPTS is the Barcelona Scoliosis Physical Therapy School. ^c^ ISST is International Schroth Three-Dimensional Scoliosis Therapy. ^d^ PSSE is physiotherapeutic scoliosis specific exercises. ^e^ n = 166 due to 7 responses missing. ^f^ n = 164 due to 9 responses missing.

**Table 2:** General characteristics of program.

	Survey n = 173 (% n of Each Category)
Familiarity with SOSORT ^a^ guidelines for AIS ^b^ [2]	
Very familiar	101 (60.8)
Somewhat familiar	44 (26.5)
I have seen them but not read them	14 (8.4)
I am not aware of the SOSORT guidelines	7 (4.2)
Combination of Treatment	
Schroth only	82 (47.5)
Combine Schroth with other PSSE ^c^	91 (52.5)
Recommend sport ^d^	
Yes, as a treatment option	4 (2.9)
Yes, as an adjunct	52 (38.2)
Yes, for general health	72 (52.9)
No, recommends non-participation	1 (0.7)
No recommendations on sport	7 (5.1)
Sport frequency ^e^	
1 time per week	9 (7)
2 or 3 times per week	100 (78.1)
4 or more times per week	19 (14.8)
Sport duration per session ^f^	
30 min or less	8 (6.3)
31–60 min	103 (80.5)
Home exercise program recommendations ^g^	
Daily	50 (38.2)
Twice daily	5 (3.8)
2 times per week	5 (3.8)
3 times per week	0 (0.0)
4 times per week	17 (13.0)
5 times per week	49 (37.4)
6 or 7 times per week	5 (3.8)
Uses a performance checklist to progress patient programs ^h^	
No	87 (65.4)
Yes	46 (34.6)
Performance checklist design (from the yes group) ^h^	
Provided to me during certification course	8/46 (17.4)
I designed it	35/44 (76.1)
Someone else designed it (outside of course)	3/46 (6.5)

^a^ SOSORT is the International Society of Scoliosis Orthopedic and Rehabilitation Treatment. ^b^ n = 166 due to 7 responses missing. ^c^ PSSE—physiotherapeutic scoliosis specific exercises. ^d^ n = 136 due to 27 responses missing. ^e^ n = 128 due to 45 responses missing. ^f^ n = 131 due to 42 responses missing. ^g^ n = 133 due to 40 missing. ^h^ n = 46 because this is from the ‘yes’ group regarding the use of a performance checklist.

**Table 3:** Frequency and duration of treatment. (173 participants) (%).

	Survey n = 173 (% n of Each Category)
Number of sessions per week treating AIS using Schroth	
<5	39 (25.5)
6–10	40 (26.1)
11–20	44 (28.8)
21–30	18 (11.8)
31+	12 (7.8)
Number of hours per week treating AIS using Schroth	
<5	34 (22.2)
5.1–10	57 (37.3)
10.1–20	34 (22.2)
20.1+	28 (18.3)
Treatment duration ^a^	
30 min	13 (8.5)
45 min	41 (26.8)
60 min	93 (60.8)
>60 min	6 (3.9)

^a^ n = 153 due to 20 missing responses.

**Table 4:** Themes, subthemes, and selected survey quotations from study interviews.

Theme 1: Adolescent as a whole Participants described various characteristics of the adolescent that influenced prescription including how there are multiple emotional and physical factors within the adolescent when prescribing Schroth and how the patient preferences were also an important factor that determined the Schroth program.	
Participants believed that emotional maturity helped with coping with diagnosis and therapy.	“[It] depends on the adolescent’s comprehension and capacity to do their correction, the breathing. …it’s not always connected with the age…it’s connected more with the maturity, cognitive maturity.” (P5, female, South America, BSPTS, 8 years experience)
Participants talked about the adolescent’s preferences influencing prescription, for example, sport.	“…I’ll actually have [the dancers] show me their dance moves. We break it down and then we talk about how much they’re going to hold their more neutral curve and be able to perform those activities as safely as possible…I work on stuff that’s more important to them.” (P4, female, North America, BSPTS, 5 years experience)
The physical characteristics and physical capacity of the adolescents	“I usually start from the floor....so it’s prone, supine and side-lying because I use gravity first to help them...afterwards when they are good in this exercise in these positions and they [sic] completely understand what they have to do in every exercise...I use gravity as an additional challenge… Depends on the curve and what their body is capable of doing. (P2, female, Europe, Both ISST and BSPTS, 5 years experience)
The personality of the child	“...if I’m too pushy, I’m afraid that they will pull back, and then we have nothing...they will pull back out of exercise... [I try] to be very careful when I talk to them...” (P2, female, Europe, Both ISST and BSPTS, 5 years experience)
Theme 2: The adolescent–parent relationship Participants highlighted how the parent–child relationship and the motivation of the parents influenced Schroth program prescription.	
Exercise prescription and attendance was aided when parents had empathy and understanding of their experience.	“I think we have to teach the parents also to see the importance of the situation and I always think about what the adolescent is going through with that.” (P6, female, South America, ISST, 2 years’ experience)
Emotional support provided by the parents.	“I have [sic] problems with the parents’ relationship with the adolescent…when I see this happening, I treat them without the parents…” (P5, Female, South America, BSPTS, 8 years’ experience)
Highly supportive parents assisted with more intense programs.	“Sometimes there are mothers who just want to have more. ‘What can we do?’” (P7, male, Europe, ISST and BSPTS, 7 years’ experience)
Theme 3: Systems Participants described how systems influenced Schroth prescription. The cost of treatment and the availability of funding influenced the frequency of sessions, and therefore, exercise prescription. Medical hierarchy was also a factor with doctor support, or lack thereof, positively and negatively affecting attendance at the clinic and referrals for X-ray assessment.	
External factors influenced prescription, especially attendance.	“I do think most people choose half-an-hour because of the benefits. Their funding through their insurance company probably covers a certain amount per visit, and so I think most people do opt for the shorter [sessions]. I have had some patients come twice a week, but very rarely. I think most don’t have the funding for that, and some have no funding so some may just come in every three weeks for half-an-hour.” (P1, female, North America, ISST and BSPTS, 3 years’ experience)
Cost of treatment, and availability of insurance, affected attendance.	“I have families who have a lot of money and the ones that [don’t]…I tell them ‘I don’t want your girls so frequently with me. I want her to learn and as soon as she learned and she’s practising at home. I will pull her in [every] two weeks or three weeks, but I want your help as parents with the compliance …Compliance on doing the exercises routinely because if this happens, I don’t need her to come to my clinic and I don’t need you to pay me anymore.’ And this is working.” (P5, female, South America, BSPTS, 8 years’ experience)
Telehealth was a useful option when distance was a factor.	“Because [named city] is a very, very large city and parents are always very busy and sometimes…they’re in another city. I have a patient now.... we are doing one session here and one by Zoom weekly. And she does exercises daily, she’s very compliant in this situation.” (P5, female, South America, BSPTS, 8 years’ experience)
COVID-19 provided a compelling reason for telehealth to be supported by insurance.	“Before the pandemic, it wasn’t allowed to do video therapy by insurance. They had to pay their own way. And in [named country] people don’t like to pay their own way. And now [we’re] allowed to do [video conferencing] …” (P7, Male, Europe, ISST and BSPTS, 7 years’ experience)
Tactile feedback from therapist was better than Telehealth for some patients.	“I had a lot of parents when all this COVID-19 situation started…a lot of parents asked me, ‘Can I do exercises or can I check their children on Zoom, like this’ and I didn’t want to do that...I see a lot more when I put my hands and fingers on the child, than when I look [on video]…” (P3, female, Europe, ISST and BSPTS, 8 years’ experience)
Medical hierarchy affected prescription and access to X-rays.	“…they booked twice a week for a long time, because the doctor said so.” (P1, female, North America, ISST and BSPTS, 3 years’ experience) “…when the patient has scoliosis and go to some doctors…most of them…tell them to wait until 45–50 degrees to do surgery. (P5, Female, South America, BSPTS, 8 years’ experience) “…because here in [country name], the physiotherapist doesn’t have the ability to ask [for imaging]. So, you have to go to the doctor to see [radiography].” (P6, Female, South America, ISST, 2 years’ experience)
Theme 4: Therapist Therapists explained how their own training and experience influenced exercise prescription.	
Therapists integrated their experience of PSSE and non-PSSE methods to adapt the program.	“I have a lot of other training and pain and biopsychosocial approach, and so I think of my training as a merger of those things, which I think is better because each patient is different...” (P1, female, North America, ISST and BSPTS, 3 years’ experience)
Therapists with other training incorporated this into Schroth program.	“So, I first try to make them familiar with breathing, with relaxing, with the mechanism, because there is breathing in Schroth but it’s easier for me to teach them through [Dynamic Neuromuscular Stabilization].” (P2, female, Europe, Both ISST and BSPTS, 5 years’ experience)
They factored in their training in other types of PSSE when they needed to progress adolescents to more dynamic exercises.	“I do a lot more SEAS. So, I get much more dynamic as they get further into the program and have a better understanding of what they need to do. So, I probably lean more heavily on Schroth work for the first four to six sessions.” (P4, female, North America, BSPTS, 5 years’ experience)
Participants highlighted how therapist experience, even before Schroth training, helped with prescription and treatment.	“… [because of my extensive experience] then for me, it’s easier to understand what is going on with the family, what is going on with the adolescent and psychological perception of what’s going on, and this helps me to make my decisions. This helps me to use something stronger or not.” (P5, female, South America, BSPTS, 8 years’ experience)

## Data Availability

Data are not available due to ethical and privacy reasons, according to the consent provided by participants.

## Appendix A. A Consensus-Based Checklist for Reporting of Survey Studies (CROSS)

**Section/Topic**	**Item**	**Item Description**	**Page Number**
Title and abstract	1a	State the word ‘survey’ along with a commonly used term in title or abstract to introduce the study’s design	2
Title and abstract	1b	Provide an informative summary in the abstract, covering background, objectives, methods, findings/results, interpretation/discussion, and conclusions.	2
Introduction Background	2	Provide a background about the rationale of study, what has been previously done, and why this survey is needed.	2
Purpose/aim	3	Identify specific purposes, aims, goals, or objectives of the study.	3
Methods Study design	4	Specify the study design in the “Methods” section with a commonly used term (e.g., cross-sectional or longitudinal).	3
Data collection methods	5a	Describe the questionnaire (e.g., number of sections, number of questions, number and names of instruments used).	5
	5b	Describe all questionnaire instruments that were used in the survey to measure particular concepts. Report target population reported validity and reliability information, scoring/classification procedure, and reference links (if any).	5
	5c	Provide information on pretesting of the questionnaire, if performed (in the article or in an online supplement). Report the method of pretesting, number of times questionnaire was pre-tested, number and demographics of participants used for pretesting, and the level of similarity of demographics between pre-testing participants and sample population.	Not performed
	5d	Questionnaire, if possible, should be fully provided (in the article, or as appendices or an online supplement).	35
Sample characteristics	6a	Describe the study population (i.e., background, locations, eligibility criteria for participant inclusion in survey, exclusion criteria).	4, 5
	6b	Describe the sampling techniques used (e.g., single stage or multistage sampling, simple random sampling, stratified sampling, cluster sampling, convenience sampling). Specify the locations of sample participants whenever clustered sampling was applied.	4, 5
	6c	Provide information on sample size, along with details of sample size calculation.	4, 5
	6d	Describe how representative the sample is of the study population (or target population if possible), particularly for population-based surveys.	23
Survey administration	7a	Provide information on modes of questionnaire administration including the type and number of contacts, the location where the survey was conducted (e.g., outpatient room or by use of online tools, such as Survey Monkey).	4, 5
	7b	Provide information of survey’s time frame, such as periods of recruitment, exposure, and follow-up days.	4
	7c	Provide information on the entry process: –>For non-web-based surveys, provide approaches to minimize human error in data entry. –>For web-based surveys, provide approaches to prevent “multiple participation” of participants.	Not performed
Study preparation	8	Describe any preparation process before conducting the survey (e.g., interviewers’ training process, advertising the survey).	Not performed
Ethical considerations	9a	Provide information on ethical approval for the survey if obtained, including informed consent, institutional review board [IRB] approval, Helsinki declaration, and good clinical practice [GCP] declaration (as appropriate).	24
	9c	Provide information about survey anonymity and confidentiality and describe what mechanisms were used to protect unauthorized access.	5
Statistical analysis	10a	Describe statistical methods and analytical approach. Report the statistical software that was used for data analysis.	5
	10b	Report any modification of variables used in the analysis, along with reference (if available).	No modifications made
	10c	Report details about how missing data were handled. Include rate of missing items, missing data mechanisms (i.e., missing completely at random [MCAR], missing at random [MAR], or missing not at random [MNAR]), and methods used to deal with missing data (e.g., multiple imputation).	5
	10d	State how non-response error was addressed.	5
	10e	For longitudinal surveys, state how loss to follow-up was addressed.	Not applicable
	10f	Indicate whether any methods such as weighting of items or propensity scores have been used to adjust for non-representativeness of the sample.	Not applicable
	10g	Describe any sensitivity analysis conducted.	Not performed
Results Respondent characteristics	11a	Report numbers of individuals at each stage of the study. Consider using a flow diagram, if possible.	4
	11b	Provide reasons for non-participation at each stage, if possible.	23
	11c	Report response rate; present the definition of response rate or the formula used to calculate response rate.	4
	11d	Provide information to define how unique visitors are determined. Report number of unique visitors along with relevant proportions (e.g., view proportion, participation proportion, completion proportion).	Not performed
	12	Provide characteristics of study participants, as well as information on potential confounders and assessed outcomes.	5
	13a	Give unadjusted estimates and, if applicable, confounder-adjusted estimates along with 95% confidence intervals and p values.	Not performed
	13b	For multivariable analysis, provide information on the model building process, model fit statistics, and model assumptions (as appropriate).	Not applicable
	13c	Provide details about any sensitivity analysis performed. If there are considerable amount of missing data, report sensitivity analyses comparing the results of complete cases with that of the imputed dataset (if possible).	Not performed
Discussion Limitations	14	Discuss the limitations of the study, considering sources of potential biases and imprecisions, such as non-representativeness of sample, study design, important uncontrolled confounders.	23
Interpretations	15	Give a cautious overall interpretation of results, based on potential biases and imprecisions and suggest areas for future research.	21–23
Generalizability	16	Discuss the external validity of the results.	21–23
Other sections			
Role of the funding source	17	State whether any funding organization has had any roles in the survey’s design, implementation, and analysis.	23–24
Conflict of interest	18	Declare any potential conflict of interest.	15
Acknowledgements	19	Provide names of organizations/persons that are acknowledged along with their contribution to the research.	Not applicable
		Based on Likert scale rating from 1 (strongly disagree) to 5 (strongly agree). Items’ scores were re-rated if major modifications were made in the previous round [10].	

## Appendix B. Consolidated Criteria for Reporting Qualitative Studies (COREQ)

**No. Item**	**Guide Questions/Description**	**Reported on Page**
Domain 1: Research team and reflexivity		
Personal Characteristics		
1. Interviewer/facilitator	Which author/s conducted the interview or focus group?	5
2. Credentials	What were the researcher’s credentials? E.g., PhD, MD	5
3. Occupation	What was their occupation at the time of the study?	5
4. Gender	Was the researcher male or female?	5
5. Experience and training	What experience or training did the researcher have?	5
Relationship with participants		
6. Relationship established	Was a relationship established prior to study commencement?	15
7. Participant knowledge of the interviewer	What did the participants know about the researcher? e.g., personal goals, reasons for doing the research	15
8. Interviewer characteristics	What characteristics were reported about the interviewer/facilitator? e.g., Bias, assumptions, reasons, and interests in the research topic	15
Domain 2: study design		
Theoretical framework		
	Developed from: [11,14].	

## Appendix C

Survey

Demographics–Personal Information

Background questions

1. Are you:
  (a) Female
  (b) Male
  (c) Prefer not to say
  (d) Other: please state
2. Age
  (a) 21–30 years
  (b) 31–40 years
  (c) 41–50 years
  (d) 51–60 years
  (e) 61+ years
3. In what professional role do you primarily treat patients (only one option is available)?
  (a) Physiotherapist/Physical Therapist
  (b) Osteopath
  (c) Exercise Physiologist
  (d) Osteopath
  (e) Chiropractor
  (f) Other–please state.
4. How long have you been working clinically in your professional role?
  (a) <1 year
  (b) 2–2.9 years
  (c) 3–4.9 years
  (d) 5–9.9
  (e) 10+ years
5. In what country are you registered to work in your primary profession (from question 3):
  (a) Australia
  (b) New Zealand
  (c) China
  (d) Taiwan
  (e) Greece
  (f) Italy
  (g) Canada
  (h) United States of America
  (i) Spain
  (j) England
  (k) Other–please specify
6. On average, how many hours per week do you work in your profession?
  (a) <10 h per week
  (b) 11–20 h per week
  (c) 21–30 h per week
  (d) 31+ hours per week
7. Which language do you primarily use to speak to patients?
  (a) English
  (b) Other–please state which language.
8. In which setting do you primarily practice your profession?
  (a) Hospital
  (b) Public setting other than hospital
  (c) Private clinic
  (d) Other–please specify

Demographics-Schroth Specific Questions

1. Are you a current member of SOSORT (The International Society of Scoliosis Orthopaedic and Rehabilitation Treatment)?
  (a) Yes
  (b) No
2. Before today, how familiar would you say you are with the ‘2016 SOSORT guidelines: orthopaedic and rehabilitation treatment of idiopathic scoliosis during growth’? Insert link to guidelines here
  (a) Very familiar.
  (b) Somewhat familiar
  (c) I have seen but not read them
  (d) I am not aware of the 2016 SOSORT guidelines.
3. Under which school or learning method have you been certified to treat scoliosis? You may choose more than one answer.
  (a) BSPTS (The Barcelona School of Physical Therapy)
  (b) ISST (International Schroth Three Dimensional Scoliosis Therapy)

Then branch off-if they choose more than one answer in question 3 they will be directed to question 4. If they only choose one answer in question 3 then they will be directed to question 5.

4. If you are certified in more than one method, choose the statement that is most applicable to you:
  (a) I only use the BSPTS method
  (b) I only use the ISST method
  (c) I use a combination of the BSPTS and ISST methods.
5. What level of scoliosis therapy education/certification have you achieved? You may circle more than one.
  (a) ISST Part 1
  (b) ISST Part 2
  (c) ISST Advanced
  (d) BSTPS C1
  (e) BSPTS C2
  (f) Other–please specify
6. In what year did you last attend an ISST or BSPTS certification or refresher?
  (a) 2018–2020
  (b) 2015–2017
  (c) 2014–2016
  (d) 2011–2013
  (e) Before 2010–state which year
7. Are you trained in other PSSE (Physiotherapeutic Scoliosis Specific Exercises) methods other than ISST or BSPTS?
  (a) Yes (go to question 8)
  (b) No (go to question 9)
8. Please state which method of PSSE other than ISST or BSPTS that you use.
9. Choose the therapies/concepts/methods that you use for your patients.

**Therapy**	**Always**	**Often**	**Sometimes**	**Rarely**	**Never**
ISST					
BSPTS					
FITS					
SEAS					
Dobomed					
Side shift					
Lyon					
Massage or soft tissue therapy					
Electrostimulation					
Pilates					
Yoga					
Other					

10. How many years have you been treating patients using the ISST and/or BSPTS method?
  (a) <1 year
  (b) 1–2 years
  (c) 3–5 years
  (d) 5–10
  (e) 11+
11. Please indicate in which groups of patients you incorporate the ISST and/or BSPTS method in your treatment? Please select all relevant responses below.
  (a) Children under 10 years of age
  (b) Adolescents (10–17.9 years)
  (c) Adults (18 + years)
  (d) Older adults 65+ years

For simplicity, from this point on we will refer to ‘Schroth’ as one method, no matter whether you use ISST, BSPTS or both. Please answer in relation to your work/therapy with Adolescent Idiopathic Scoliosis (AIS) patients only.

12. For how many years have you been incorporating Schroth exercises in your treatment of patients with Adolescent Idiopathic Scoliosis (AIS)?
  (a) <1 year
  (b) 2–3 years
  (c) 3–5 years
  (d) 5–10
  (e) 10–20
13. On average, how many initial assessments for adolescents with idiopathic scoliosis would you do in a week?
  (a) <5 sessions per week
  (b) 6–10 sessions per week
  (c) 11–20 sessions per week
  (d) 21–30 sessions per week
  (e) 31+ sessions per week.
14. On average, how many Schroth treatment sessions for adolescents with idiopathic scoliosis would you do in a week? (Total number of the year/52weeks per year).
  (a) <5 sessions per week
  (b) 6–10 sessions per week
  (c) 11–20 sessions per week
  (d) 21–30 sessions per week
  (e) 31+ sessions per week.
15. Would any of these sessions be group sessions?
  (a) Yes (go to question 15 and 16)
  (b) No (go to question 17)
16. What percentage of your total Schroth treatment sessions per week are group sessions?
  (a) <10%
  (b) 11–25%
  (c) 26–50%
  (d) 51–75%
  (e) 76–99%
  (f) 100%
17. What is the average ratio of practitioner to patient for these group sessions?
  (a) 1:2
  (b) 1:3
  (c) 1:4
  (d) 1:5
  (e) Other–please specify
18. Choose the most appropriate statement that reflects your work situation a majority of the time with patients with Adolescent Idiopathic Scoliosis (AIS):
  (a) I treat patients with AIS less than 5 h per week.
  (b) I treat patients with AIS between 5.1 and 10 h per week.
  (c) I treat patients with AIS between 10.1 and 20 h per week
  (d) I treat patients with AIS more than 20.1 h per week.
19. How long, on average, would your initial assessment sessions with your patients be? Choose the closest answer.
  (a) 30 min
  (b) 45 min
  (c) 60 min
  (d) Other–please specify
20. How long, on average, would your treatment sessions with your patients be? Choose the closest number.
  (a) 30 min
  (b) 45 min
  (c) 60 min
  (d) Other–please specify
21. What percentage of your total patients have adolescents with Idiopathic Scoliosis?
  (a) <10% (no further questions)
  (b) 11–20%
  (c) 21–50%
  (d) 51–80%
  (e) 81–100%
22. Do you work in a multidisciplinary team when treating adolescents with Idiopathic Scoliosis with the Schroth method?
  (a) Yes (go to question 22)
  (b) No (go to question 23)
23. Who is a member of your multidisciplinary team? (Choose all that apply)
  (a) Physiotherapists (Physical Therapists)
  (b) Chiropractors
  (c) Osteopaths
  (d) Doctors (General Practitioners)
  (e) Spinal Surgeons
  (f) Orthotists
  (g) Psychologists
  (h) Nutritionist/Dietitian
  (i) Exercise Physiologist
  (j) Other–please describe
24. Are you responsible for deciding which exercises your patients with AIS do?
  (a) Yes (go to question 25)
  (b) No (go to question 24)
25. Who is responsible for deciding which exercises your AIS patients do?
  (a) A doctor/medical practitioner not specialized in spinal conditions
  (b) A doctor/medical practitioner that specializes in spinal conditions
  (c) Other medical or allied health professional–please state the role of this person (no names).
  (d) Other–please specify.
26. How effective do you think the Schroth method is in regard to treating AIS? Rate your answer from 0–10 (0 being not effective at all, and 10 being extremely effective)?

Provide a scale from 0–10

Exercise questions

For the following questions please answer in relation to your work as a Schroth therapist treating patients with Adolescent Idiopathic Scoliosis (AIS).

1. When do you first commence implementing Schroth for your patients?
  (a) At the initial assessment
  (b) At the first therapy session
  (c) Within the first month of therapy sessions (go to question 2)
  (d) After the first month of therapy sessions (go to question 2)
  (e) Other–please specify (go to question 2)
2. What type of therapy do you implement before starting Schroth? You may choose more than one answer.
  (a) Lyon
  (b) SEAS
  (c) FITS
  (d) Side Shift
  (e) Dobomed
  (f) Other–please specify
3. How often do you see most of your patients in a face-to-face setting in the first week after the initial assessment?
  (a) 0
  (b) 1
  (c) 2
  (d) 3
  (e) 4 or more times
4. How often do you see most of your patients in a face to face setting in the first month after the initial assessment?
  (a) 0
  (b) 1–3 times
  (c) 4–6 times
  (d) 7–10 times
  (e) 11–24 times
  (f) Other–please specify
5. How often do you see most of your patients in a face-to-face setting in the first three months (12 weeks) after the initial assessment?
  (a) 0
  (b) 1–3 times
  (c) 4–6 times
  (d) 7–10 times
  (e) 11–24 times
  (f) Other–please specify
6. Please fill in the following table in regard to how often you implement different aspects of therapy for AIS.

	**Always**	**Often**	**Sometimes**	**Rarely**	**Never or Not Applicable**
I use autocorrection in 3D (three dimensions)					
I implement training in activities of daily living (ADLs)					
I implement strategies to stabilize the corrected posture					
I provide patient education in sessions					
I use axial expansion techniques					
I use pelvic corrections in the exercises					
I use verbal cues					
I use tactile (touch) cues					
I use a mirror to provide patients feedback					

7. Please fill in the table in relation to how often you incorporate the following exercise characteristics in your treatment sessions.

**Exercise**	**Always**	**Often**	**Sometimes**	**Rarely**	**Never, But I Am Familiar with Schroth Exercise/s with This Characteristic.**	**Never, But I Am Not Familiar with Any Schroth Exercise with This Characteristic**
Exercises that incorporate psoas activation						
Exercises sitting on a ball						
Exercises standing between two poles						
Exercises sitting between two poles						
Exercises with a foot or knee under a bar (for resistance or stability)						
St. Andrew’s Cross						
Muscle cylinder						
Schroth walking						
Exercises in a supine position						
Exercises in a Side lying position						
Exercises in a Prone position (on knees or stool)						
Full hanging						
Semi hanging						
Supported hanging						

8. Do you adjust the difficulty of the Schroth exercises for each patient?
  (a) Yes (go to question 9)
  (b) No (go to question 10)
9. Please fill in the following table regarding how you adjust the exercises?

	**Always**	**Often**	**Sometimes**	**Rarely**	**Never**
I encourage the increase or decrease of the intensity of vector forces coming from the patient, for example by expansion techniques					
I add or remove resistance, e.g., weights or bands					
I add in or remove sticks or other props					
I add in or remove unstable surfaces, e.g., fitball or wobble board					
I increase or decrease the required repetitions					
I increase or decrease the duration of the Schroth exercises					
I add or remove passive support.					

10. Please fill in the table below as to how often the following factors affect your choice of exercise for your patients.

**Factor**	**Always**	**Often**	**Sometimes**	**Rarely**	**Never**
Age of the child					
Stage of growth and growth remaining, Risser sign or Sanders Score					
Stiffness of the curve, e.g., as defined by ability for hump to reduce in size with particular movements.					
The presence of hypermobility, e.g., Beighton’s score 6 or more out of 9					
The presence of muscle tightness, e.g., hamstring straight leg raise <90 degrees or positive Thomas test					
Size of the curve/s					
Location of the curve/s, e.g., thoracic versus lumbar curve					
Presence of more than one curve					
The presence of behavioral concerns, e.g., related to ADHD (Attention Deficit Hyperactive Disorder)					
The presence of psychological concerns					
Financial considerations					
The presence of spondylolisthesis					
The presence of pain					
Other–please explain					
Other–please explain					
Other–please explain					

11. Please fill in the table below in relation to how often the following factors influence the frequency of patient attendance at the clinic:

	**Always**	**Often**	**Sometimes**	**Rarely**	**Never**
Location/vicinity to the clinic (ease of access)					
Financial constraints of the family					
Healthcare support in your country					
Mental health status of the child					
Ability of the child to concentrate.					
Ability of the child to perform the exercises.					
Motivation of the child					
Motivation of the parents/guardian					
COVID-19					
Other–please explain.					
Other–please explain.					
Other–please explain.					

12. Please fill in the table below to indicate how frequently you prescribe the following exercises or features in your programs.

	**Always**	**Often**	**Sometimes**	**Rarely**	**Never**
Static, non-weight bearing, no props					
Static, non-weight bearing, with props					
Dynamic, non-weight bearing, no props					
Dynamic, non-weight bearing, with props					
Wall bar					
Sticks or props to facilitate pressure					
Patient handouts for explanations					
Audio recordings					
Video recordings					
Photos/diagrams					
Breathing techniques					

13. Do you include any manual therapy when treating your patients? If yes, what type (you can choose more than one answer):
  (a) Mobilisations of the spine
  (b) Manipulations of the spine
  (c) Soft tissue release/massage
  (d) Static stretches
  (e) Dynamic stretches
  (f) Other–please specify
  (g) No I don’t use manual therapy
14. Do you recommend sport as part of treatment *?
  (a) Yes I recommend it as a treatment option. (go to question 15)
  (b) Yes I recommend it as an adjunct to Schroth treatment. (go to question 15)
  (c) Yes I recommend sport for general health reasons only. (go to question 15)
  (d) No, I recommend that they do not participate in sport (go to question 17)
  (e) No, I don’t provide any recommendations regarding sport at all (go to question 17).

* Sport may include active individual or team pursuits, including but not limited to tennis, football, dance, gymnastics etc.

15. How many times per week do you recommend your patients do sport?
  (a) 1 time
  (b) 2 or 3 times per week
  (c) 4 or more times per week

* Sport may include active individual or team pursuits, including but not limited to tennis, football, dance, gymnastics etc.

16. How much time do you recommend that they should participate in sport during each sport session *?
  (a) 30 min or less
  (b) 31–60 min
  (c) 61 min or more

* Sport may include active individual or team pursuits, including but not limited to tennis, football, dance, gymnastics etc.

17. Do you believe that any specific sport is helpful for scoliosis?
  (a) yes (go to question 18)
  (b) no (go to question 19)
18. Which sport do you think is the most helpful for patients with scoliosis?
  Please state
  Then proceeds to question 19

19. Do you believe that any specific sport has a negative effect on scoliosis?
  (a) yes (go to question 20)
  (b) no (go to question 21)
20. Which sport do you think has a negative effect on scoliosis?
  (a) Please state
  (b) Then proceeds to question 21.
21. How often do you include or encourage the following in your exercise programs that you prescribe for adolescents with AIS?

	**Always**	**Often**	**Sometimes**	**Rarely**	**Never, But I Am Familiar with This Exercise.**	**Never, But I Am Not Familiar with This Exercise**
Pilates						
Yoga						
Strength training using weights						
Strength training using functional exercise (exercises that use most of the body)						
Flexibility exercise						

22. Do you provide a home exercise program?
  (a) Yes (go to question 23)
  (b) No (go to question 27)
23. Please specify the average frequency of exercise participation that you recommend for their home exercise program?
  (a) Daily
  (b) Twice daily
  (c) 2 times per week
  (d) 4 times per week
  (e) 5 times per week
  (f) Other–please specify

Then proceed to question 24.

24. Please specify the average duration of exercise that you recommend for each home program?
  (a) 5–10 min per day
  (b) 11–20 min per day
  (c) 21–30 min per day
  (d) 31–45 min per day
  (e) 46–60 min per day
  (f) 61 min + Specify __________

Then proceed to question 25.

25. How many Schroth exercises do you give your patients to practice at home in the first week?
  (a) 1
  (b) 2–3
  (c) 4–5
  (d) Other–please specify

Then proceed to question 26.

26. How many Schroth exercises do you give your patients to practice in clinic and home in the 1–3 months
  (a) 1
  (b) 2–3
  (c) 4–5
  (d) Other–please specify
27. Do you use an exercise performance checklist * before progressing patient programs?
  (a) Yes (go to question 28)
  (b) No (go to question 29)

* A list of criteria to establish whether you are satisfied that the patient’s performance in the program.

28. Who designed this exercise performance checklist* ?
  (a) It was provided to me during my certification course. (go to question 30)
  (b) I designed it. (go to question 30)
  (c) Someone else designed the checklist outside of my certification course (go to 29).
29. Who designed it?
  (a) Another Schroth therapist
  (b) A non-Schroth therapist–please state the profession of this person or persons (no names please).
30. To what extent do you follow the Schroth protocol or teachings you were taught?
  Add sliding scale:
  0–never, I use other methods
  5–50% of the time and 50% of the time I use other methods
  10–All the time

31. How frequently, if at all, do you monitor how your patients do or don’t follow your exercise advice or instructions using the following?

	**Always**	**Often**	**Sometimes**	**Rarely**	**Never**
a logbook					
an App					
Other–please state					

32. Would you like to receive further information regarding follow-up interviews to further explore this topic in more depth? Your identity will not be disclosed in the publication of results.
  (a) yes—thank you! This will then link to a short additional survey. [see below]
  (b) no—thank you. No further questions.

Thank you for your time! If you would like to receive a summary of results, please email rosemary.marchese@hdr.mq.edu.au.

Short Survey for Participants from Part 1 who answer ‘yes’ to question 32.

Participants who have chosen to receive information regarding follow-up inter-views to further explore this topic will be directed to another survey with the following questions.

1. Please state your full name.
2. What is your email address? (please check this is correct)
3. What is your nationality?
4. In what country do you currently use the Schroth method?
5. How many years’ experience do you have using the Schroth method for AIS?

## Appendix D. Semi-Structured Interview Questions

Note: No non-participants were present during the semi-structured interviews.

Aim: To determine the factors that influence Schroth therapists’ decisions when prescribing Schroth therapy to patients.

Questions were refined once the survey results were received and analysed. This updated version of the interview questions was submitted in an amended ethics application and approved. The semi-structured interview was recorded on Zoom (camera on) by RM and took place at a time suitable for the participant. The recording was saved on Macquarie University Office 365 account. The interview was transcribed line by line, checked by RM, EI, and the individual participant, and saved on Macquarie University Office 365 account.

The interviews each took approximately 45 to 60 min.

The Interview

‘Thank you for participating today, [insert interviewee name]. My name is Rosemary Marchese and I will be conducting this interview today. The purpose of the interview is to explore factors influencing your decisions when prescribing Schroth therapy to adolescents with idiopathic scoliosis. You can stop the interview at any time, and you are also encouraged to stop and ask questions or seek further clarification. You will be given a simple case study that we will discuss during the interview.

This interview is being recorded for research purposes. Are you happy to continue?

Case study: Imagine this scenario in your clinic. An adolescent child attends with their family and would like to start Schroth therapy for idiopathic scoliosis.

[Note: Details of the case study have been minimised on purpose to maximise the opportunity for the participant to ask questions and for the importance of those questions to be explored by the Interviewer.]

Questions:

1. To determine your exercise prescription for this adolescent, what further information would you want to know before commencing treatment?
2. Why are those factors important to consider?

Example: If the interviewee asks the child’s age, then the Interviewer can ask the interviewee to explain why age is important.

1. Tell me about your typical choice of Schroth exercises in your first week of treatment for this patient.
2. Tell me about your typical choice of Schroth exercises once the patient is more experienced, say after two or three months of treatment.
3. Would there be any Schroth exercises that you would prefer or any that you would avoid, for this patient, and why?
4. Have you ever modified a Schroth exercise from the original training you received, and if so how and why?
5. Are there any Schroth exercises that you believe are harmful to the patient and why?
6. Would your own professional experience and personal circumstances influence your treatment of this patient or your Schroth career? How?

Probing questions may include:

1. What factors influence your decisions in regard to the number of exercises you prescribe for an adolescent with idiopathic scoliosis (AIS)?
2. Explain the personal factors within the child and their situation that influences your exercise prescription.
3. If the curve was bigger/smaller what would change in your approach?
4. If the child was younger/older what would change in your approach?
5. What factors influence your decision in regard to the type of exercises you prescribe for patients with AIS?
6. Does the adolescent’s personal situation, for example, vicinity to the clinic, finances, family support, influence your prescription? If so, how?
7. Does the adolescent’s medical or health situation, for example size of curve, Risser sign, use of a brace, influence your prescription? If so, how?
8. Do the risk factors for progression of a curve influence your therapy prescription? If so, how?
9. (a) Explain how you try to keep a child motivated.
  (b) Do you think this is successful?
  (c) Why or why not?
10. How do you decide where to start when prescribing exercises to an adolescent with Idiopathic Scoliosis?
11. How do you develop the plan for the adolescent with idiopathic scoliosis? Hint if necessary: Do the family and patient collaborate with you to establish the plan? Do you recommend a particular dosage for all? Are you directed by someone else to make decisions?
12. How do you decide which Schroth exercises to prescribe?
13. How does your Schroth training influence the dosage or therapy that you prescribe for your patients?
14. How closely do you stick to what you were taught versus what you actually do?

Thank you for your participation in this interview. Do you have any final questions or comments for me?

This interview will be transcribed line by line. I will email you the transcription for you to check. You will have a week to respond with any comments that you may have.

Thank you for your time.

Then close the interview.
